# Role of HK2 in the Enzootic Cycle of *Borrelia burgdorferi*

**DOI:** 10.3389/fmed.2020.573648

**Published:** 2020-10-26

**Authors:** Qiang Liu, Haijun Xu, Yan Zhang, Jing Yang, Jimei Du, Yan Zhou, X. Frank Yang, Yongliang Lou

**Affiliations:** ^1^Wenzhou Key Laboratory of Sanitary Microbiology, Key Laboratory of Laboratory Medicine, Ministry of Education, School of Laboratory Medicine and Life Sciences, Wenzhou Medical University, Wenzhou, China; ^2^Department of Microbiology and Immunology, Indiana University School of Medicine, Indianapolis, IN, United States; ^3^State Key Laboratory of Rice Biology and Ministry of Agriculture Key Laboratory of Agricultural Entomology, Institute of Insect Sciences, Zhejiang University, Hangzhou, China; ^4^Optometry and Eye Hospital and School of Ophthalmology, School of Biomedical Engineering, Wenzhou Medical University, Wenzhou, China

**Keywords:** lyme disease, *Borrelia* (Borreliella) *burgdorferi*, two-component system (TCS), Rrp2-HK2, OspC

## Abstract

The two-component response regulator Rrp2 is a key activator controlling the production of numerous virulence factors of *Borrelia burgdorferi*, the Lyme disease pathogen. Previously it was shown that the cognate histidine kinase HK2 is not required for Rrp2 activation *in vitro*, nor for mammalian infection upon needle inoculation, raising the question whether HK2 has any role in the enzootic cycle of *B. burgdorferi*. In this study, we demonstrated that HK2 is not required for spirochetal survival in the tick vector. When fed on naive mice, the *hk2* mutant had reduced infectivity through the route of tick bite, suggesting that the spirochetes lacking HK2 had a disadvantage in the enzootic cycle. Furthermore, overexpression of *hk2* reduced the level of Rrp2 phosphorylation, suggesting that HK2 can function as a phosphatase to dephosphorylate Rrp2. Strains overexpressing *hk2* impaired the expression of RpoN regulon whose activation is dependent on Rrp2 phosphorylation and activation, and had reduced infectivity in mice. Taken together, these results demonstrate that although HK2 does not play an essential role in Rrp2 activation, it is important for the optimal fitness of *B. burgdorferi* in the enzootic cycle.

## Introduction

The Lyme disease pathogen, *Borrelia burgdorferi, B. afzelii*, and *B. garinii*, is maintained in nature in two drastic different hosts, an *Ixodes* tick and a mammalian host. As an obligate parasite with a small genome, *B. burgdorferi* has evolved using its limited signaling and regulatory repertoire to adapt to both host environments. Comparing to free living bacteria such as *Escherichia coli*, which has more than 30 two-component signal transduction systems (TCSs), *B. burgdorferi* has reduced to two sets of TCS, HK1/Rrp1 and HK2/Rrp2 (in addition to the chemotactic CheA-CheY system) and has evolved to employ these two TCSs to survive in each of the two hosts encountered in the enzootic cycle. HK1/Rrp1, a c-di-GMP producing system, controls spirochete's adaptation to the tick vector (1–7), whereas HK2/Rrp2 is essential for *B. burgdorferi* to establish infection in the mammalian host (8–11).

The function of HK2/Rrp2 is largely based on the study of the response regulator Rrp2. A typical TCS consists of a histidine kinase as a sensor and a corresponding response regulator that mediates the cellular response (12). Rrp2 is a member of NtrC family transcriptional activator. It contains three putative functional domains: an N-terminal response regulator receiver domain, a central activation domain, and a C-terminal helix-turn-helix (HTH) DNA-binding domain (38). The central activation domain of Rrp2 is highly conserved among a group of transcriptional activators (bacterial enhancer-binding proteins) that specifically activate genes from alternative sigma factor 54 (RpoN or σ^54^)-type promoter. The only gene with a σ^54^-type promoter in *B. burgdorferi* identified to date is *rpoS*, which encodes the second alternative sigma factor RpoS (σ^S^). Thus, upon activation by phosphorylation at its N-terminal receiver domain, Rrp2 activates its central domain, leading to activation of an alternative sigma factor cascade, σ^54^-σ^S^ cascade (also called RpoN-RpoS pathway or Rrp2-RpoN-RpoS pathway) in *B. burgdorferi* [for review, see (13–15)]. Rrp2, along with a transcriptional activator BosR and a repressor BadR, regulates σ^54^-σ^S^ sigma factor cascade, which in turn controls *ospC, dbpB/A, bbk32*, and many other mammalian infection-associated genes (8–11, 16–26).

Relative to the downstream targets controlled by Rrp2, the upstream event that activates Rrp2 is poorly understood. The gene *hk2* (BB0763) is adjacent to *rrp2* (BB0764) in the genome and purified HK2 is capable of phosphorylating Rrp2 *in vitro*, making HK2 qualified as the cognate histidine kinase for Rrp2 (8, 27). However, we and others previously showed that disruption of *hk2* does not affect *ospC* expression and activation of σ^54^-σ^S^ cascade, suggesting that HK2 is not essential for Rrp2 phosphorylation (27, 28). The *hk2* mutant is also capable of infecting mice upon needle infection. This raises question whether HK2 plays any role in the enzootic cycle of *B. burgdorferi*. In this study, we further investigated the *hk2* mutant phenotype in the tick phase of the enzootic cycle, showing that the *hk2* mutant had a reduced infection via tick infestation. We also took another approach to study HK2 functions by overexpressing *hk2*. The results showed that HK2 is functional and important for maximum fitness in the enzootic cycle of *B. burgdorferi*.

## Results

### HK2 Is Not Required for Spirochete Survival During Tick Feeding

**Figure 1:**
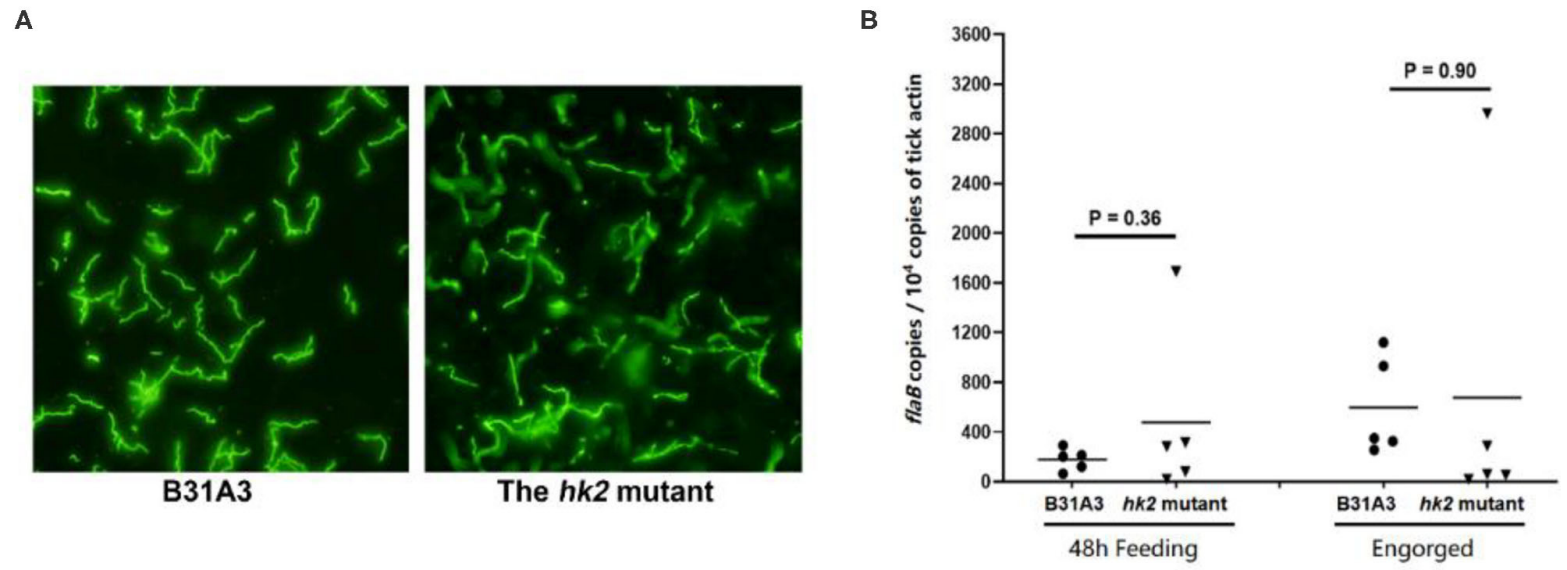
IFA and qPCR analyses of spirochetes in fed larvae. **(A)** Unfed *I. scapularis* larvae harboring either the wild type B31A3 or the *hk2* mutant were fed on naive C3H/HeN mice, and engorged larvae were subjected to IFA analysis using fluorescein isothiocyanate-labeled anti-*B. burgdorferi* antibody. Five ticks were examined for each group, and a representative image for each group of ticks is shown. **(B)** 48 h feeding larvae or fully fed larvea were collected and subjected to qPCR analyses. Each data points were generated from the DNA sample extracted from three larvae. The copy numbers of the *flaB* gene of *B. burgdorferi* was used for caculating spirochetal numbers, which is nurmalized by 10^4^ copies of the tick actin gene.

Previously we showed that the *hk2* mutant had normal infectivity in mice upon needle inoculation (28). To examine whether HK2 plays a role in tick phase of the enzootic cycle, naive *Ixodes scapularis* larvae were fed on immunocompetent C3H/HeN mice that were needle-infected with wild-type or the *hk2* mutant spirochetes. During and after depletion, engorged larvae were then subjected to immunofluorescent assay (IFA) (Figure 1A) and qPCR analyses (Figure 1B) to assess the spirochetal numbers. The result showed that there was no significant difference in spirochetal numbers in the tick midguts at 48 h during feeding or after feeding on mice infected with wild-type and the *hk2* mutant *B. burgdorferi*, suggesting that HK2 is not required for spirochetal survival during tick feeding (Figures 1A,B).

### The *hk2* Mutant Has Normal Level of Activation of σ^54^-σ^S^ Sigma Factor Cascade During Tick Feeding

**Figure 2:**
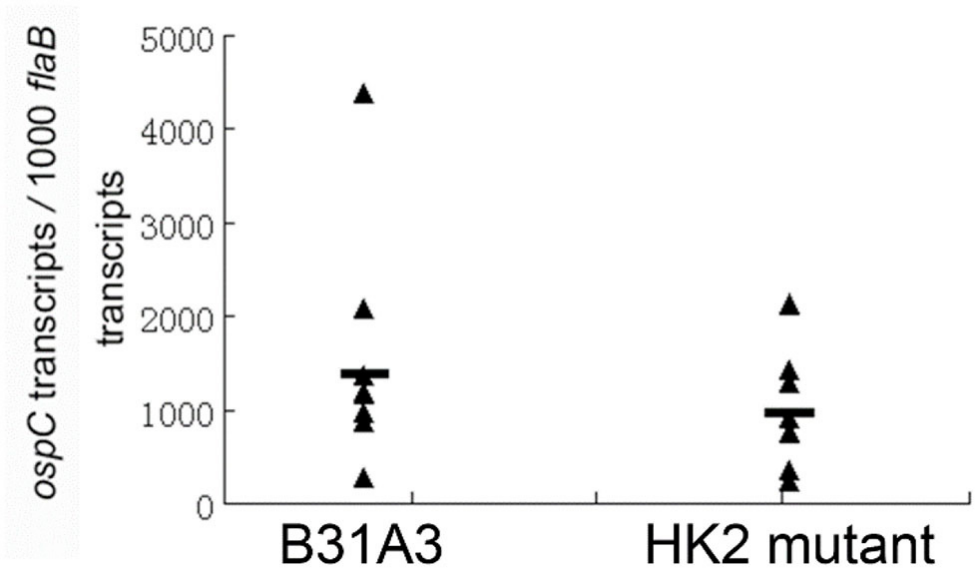
qRT-PCR analyses of spirochetes during tick feeding. Flat *I. scapularis* nymphs infected with either the wild-type strain B31A3 or the *hk2* mutant were fed on naive C3H/HeN mice, and engorged nymphs were subjected to qPCR analyses. Seven ticks were examined for each group, and each data point was from one nymph. The level of *ospC* expression were nornalized with the level of *flaB* transcripts of *B. burgdorferi*. No significant difference was observed between the two groups.

We and others showed that HK2 is not required for Rrp2 activation and σ^54^-σ^S^ cascade activation under the *in vitro* cultivation conditions (27, 28). In the enzootic cycle of *B. burgdorferi*, spirochetes colonizing in the midgut of unfed ticks begin to replicate and activate σ^54^-σ^S^ cascade when ticks feed (14). Whether HK2 is required for σ^54^-σ^S^ cascade activation during tick feeding has not been examined. Thus, infected fed larvae from above experiments were allowed to molt to nymphs. Flat nymphs were fed on naive mice. Engorged nymphs were collected for quantitative RT-PCR analysis to determine the levels of ospC expression, a surrogate for σ^54^-σ^S^ cascade activation. As shown in Figure 2, there was no significant difference in levels of ospC expression between wild-type spirochetes and the *hk2* mutant during tick feeding, suggesting that HK2 is not required for σ^54^-σ^S^ cascade activation *in vitro* as well as during tick feeding.

### The *hk2* Mutant Has Reduced Infectivity via Tick Infestation

Although HK2 is not required for mammalian infection via needle inoculation using *in vitro* cultured spirochetes, it remains to be determined whether HK2 plays a role via tick infestation. Accordingly, flat nymphs harboring wild-type or the *hk2* mutant spirochetes were allowed to feed on groups of naive C3H/HeN mice. Three weeks after tick bites, mice were sacrificed, and tissue biopsies including skin, joint, and heart were collected for culturing the presence of spirochetes. While 86.7% of mouse tissues infected with ticks harboring wild-type *B. burgdorferi* were culture positive, 48.3% mouse tissues infected with ticks harboring the *hk2* mutant were culture positive (Table 1), suggesting that the *hk2* mutant has reduced infectivity via the route of tick infection.

**Table 1:** The infectivity of the *hk2* mutant via tick bite^¶^.

**Strains**	**No. of mouse tissues culture positive/total No. of tissues tested**			**No. of tissues infected/total No. of tissues**
	**Skin**	**Joint**	**Heart**	
WT (B31A3)	13/15	13/15	13/15	39/45 (86.7%)*
The *hk2* mutant	8/20	11/20	10/20	29/60 (48.3%)*

¶ Dose of infection: 5 nymphs per mouse.

* *The p-value between the two group is 0.04 (Fisher's Exact Test)*.

### Overexpression of *hk2* Impairs Activation of σ^54^-σ^S^ Sigma Factor Cascade

**Figure 3:**
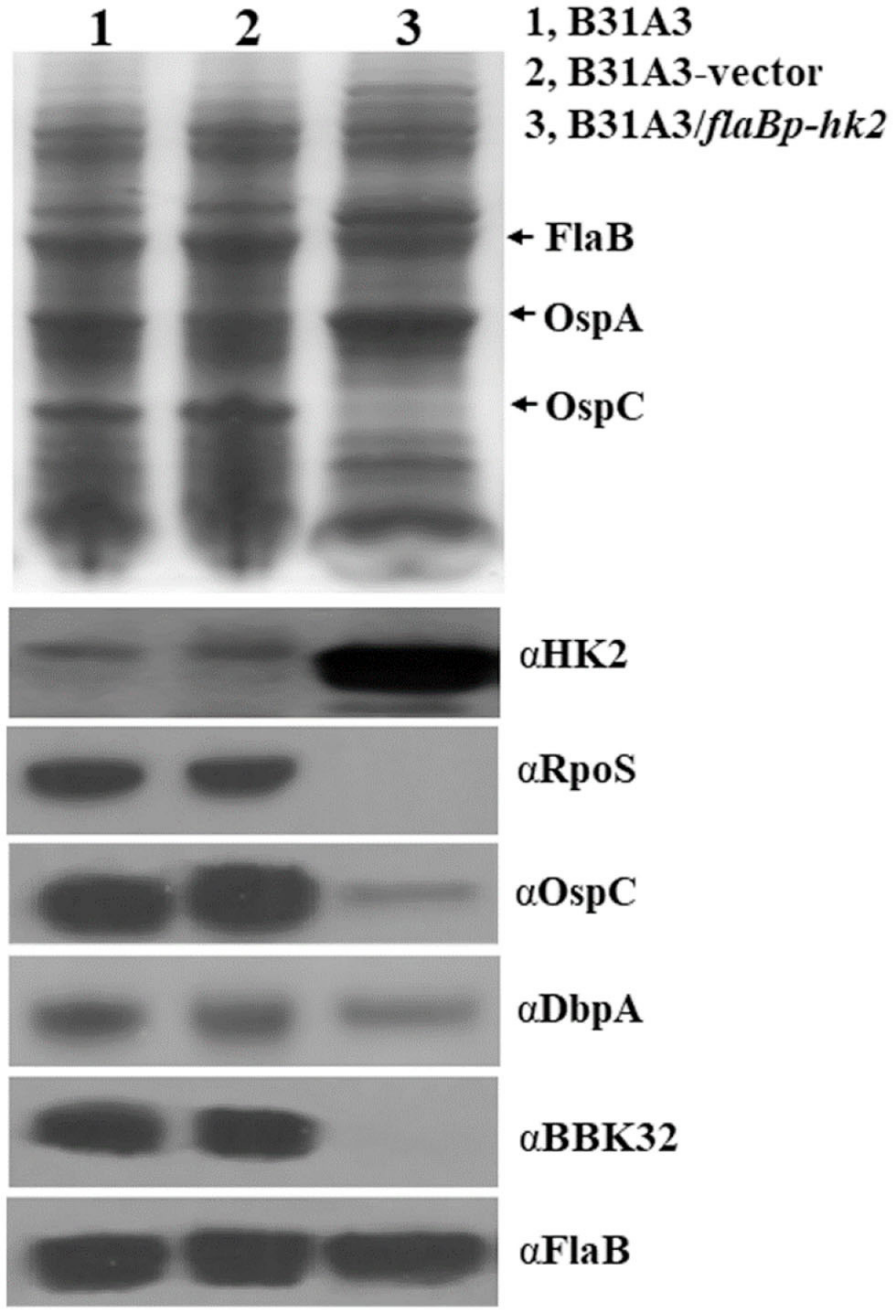
Overexpression of HK2 impaired production of RpoS and RpoS-controlled lipoproteins. Spirochetes were cultivated in BSK-II medium at 37°C and were harvested at the stationary phase, and whole-cell lysates were subjected to SDS-PAGE (top) and Western blot analyses (bottom). The positions of proteins and antibodies used are indicated on the right. B31A3, wild-type *B. burgdorferi*; B31A3-vector, B31A3 carrying an empty shuttle vector; B31A3/*flaBp*-HK2, B31A3 carrying a vector harboring a *hk2* gene driven by a constitutive *flaB* promoter.

**Figure 4:**
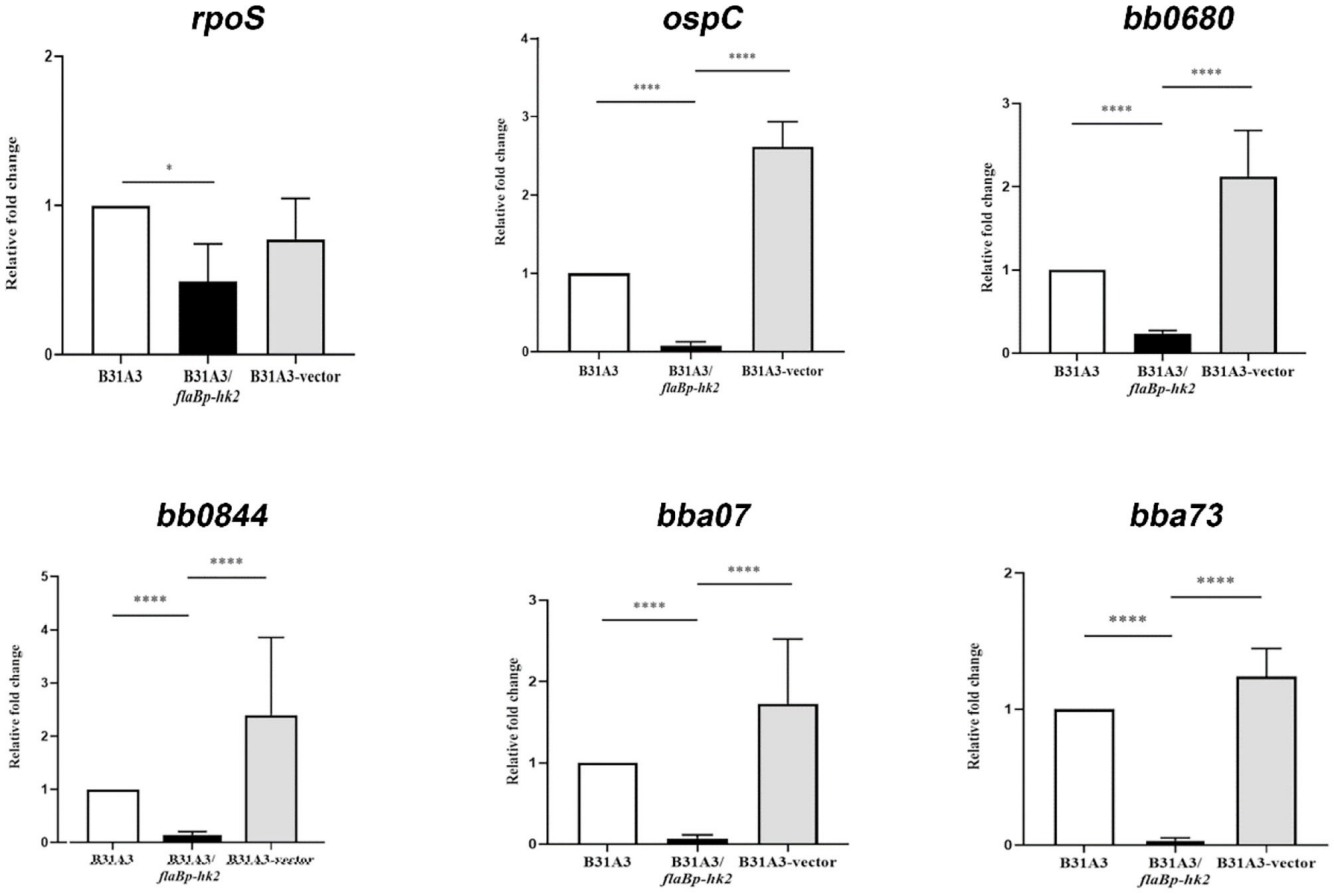
qRT-PCR analyses of several RpoS-controlled genes. Spirochetes were cultured at 37°C in BSK-II medium and harvested in the late-log phase. RNAs were extracted and subjected to qRT-PCR analyses. Samples were first normalized with the *flaB* level, and then levels of gene expression were reported relative to that of wild-type B31A3 (with the level of expression of each gene in B31A3 as 1.0). *p < 0.01; ****p < 0.0001.

To further investigate the function of HK2, we took another approach by overexpressing *hk2* by transforming *B. burgdorferi* with a shuttle vector carrying a *hk2* gene driven by a constitutive *flaB* promoter. The shuttle vectors were transformed into wild-type strain B31A3. As expected, transformed clones carrying a *flaB* promoter-driven *hk2* had a much higher level of Hk2 than that of wild-type strain (Figure 3). Overexpression of *hk2* dramatically reduced production of RpoS and RpoS-dependent surface lipoproteins such as OspC, DbpA, and BBK32 (Figure 3). Further qRT-PCR analyses showed that transcripts of *rpoS* and several RpoS-dependent genes (*ospC, bb0680, bb0844, bba07, bba73*) were significantly reduced upon HK2 overexpression (Figure 4). These results indicate that overexpression of *hk2* impaired the activation of σ^54^-σ^S^ cascade.

### Overexpression of *hk2* Reduces Infectivity

Given the importance of σ^54^-σ^S^ cascade in mammalian infection, we examined the ability of the *hk2* overexpression strain to infect mice. Groups of immunocompetent C3H/HeN mice were inoculated with either a high dose (1 × 10^5^ spirochetes per mouse) or a low dose (1 × 10^3^ spirochetes per mouse) of wild-type *B. burgdorferi* B31A3 or the *hk2* overexpression strain B31A3/*flaBp-hk2*. Four weeks post-inoculation, mice were sacrificed, and various mouse tissues (skin, heart, and joint) were collected and cultured for spirochete growth. All mouse tissues from mice inoculated with wild-type *B. burgdorferi* B31A3 were culture positive, whereas mouse tissues from mice inoculated with B31A3/*flaBp-hk2* were 33% (*p* = 0.16) and 19% (*p* = 0.01) culture positive for high dose group and low dose group, respectively (Table 2), indicating that overexpressing *hk2* reduced the infectivity of *B. burgdorferi* in mice.

**Table 2:** The infectivity of the *hk2* overexpression strain.

***Borrelia* strains and infection dose**	**No. of mouse tissues culture positive/total No. of tissues tested**			**No. of tissues infected/total No. of tissues**
	**Skin**	**Joint**	**Heart**	
1 × 10^5^/mouse				
B31A3	3/3	3/3	3/3	9/9 (100%)
B31A3/*flaBp-hk2*	2/3	0/3	1/3	3/9 (33%)*
1 × 10^3^/mouse				
B31A3	7/7	7/7	7/7	21/21 (100%)
B31A3*/flaBp-hk2*	0/7	4/7	0/7	4/21 (19%)^§^

* The p-value between the two groups is 0.16.

§ *The p value between the two groups is 0.01*.

### HK2 Functions as a Phosphatase of Rrp2

**Figure 5:**
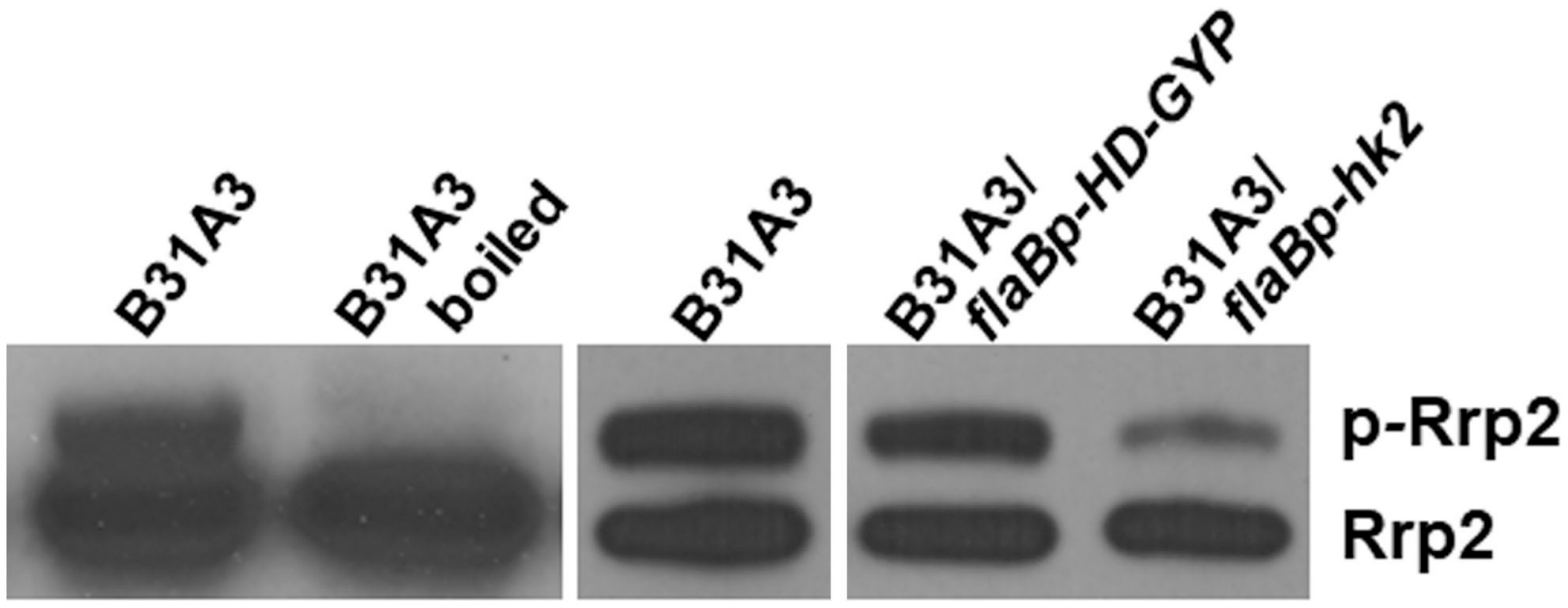
Overexpressing HK2 reduces the level of phosphorylated Rrp2 in *B. burgdorferi*. Phos-tag SDS-PAGE and immunoblotting was used to detect both phosphorylated and dephosphorylated Rrp2 in the cell. Wild-type *B. burgdorferi* B31A3, B31A3 carrying a shuttle vector harboring a unrelated protein HD-GYP (B31A3*/flaBp-HD-GYP*), or B31A3 carrying a shuttle vector harboring a *hk2* gene driven by a *flaB* promoter GYP (B31A3*/flaBp-hk2*), were harvested at mid-log phase and cell lysates were prepared and separated on 7.5% SDS-PAGE containing 0, 5, 10, and 25 uM Phos-tag followed by immunoblotting using anti-Rrp2 antibody. p-Rrp2, the band corresponds to phosphorylated Rrp2. As a unphosphorylated Rrp2 control, B31A3 was also treated by boiling (lane 2) prior to Phos-tag SDS-PAGE (Rrp2 phosphorylation is unstable and sensitive to heat).

We further investigated possible mechanisms underlying the phenotypes of HK2 overexpression. Some two-component histidine kinases can function as phosphatase (29, 30). Given that activation of σ^54^-σ^S^ cascade requires Rrp2 phosphorylation, we postulate that Hk2 may function as a phosphatase for Rrp2. Because aspartate phosphorylation of response regulators has short half-life and is very unstable, and antibodies that recognize phospho-Asp are not available, we performed Phos-tag™ acrylamide gel electrophoresis that uses dinuclear metal complex as a specific phosphate-binding agent to chelate phosphate (31, 32), a method that has shown to successfully separate phosphorylated and unphosphorylated forms of other response regulator proteins (33). Accordingly, *B. burgdorferi* lysates were subjected to Phos-tag SDS-PAGE followed by immunoblotting using anti-Rrp2 monoclonal antibody. Two distinct bands were observed in B31A3 (Figure 5, lane 1): the lower band corresponded to unphosphorylated Rrp2 and the top band corresponded to phosphorylated Rrp2 (p-Rrp2). When the cell lysate was treated by heat (boiling) prior to Phos-tag SDS-PAGE, the top band disappeared (Figure 5, lane 2), consistent with the fact that Asp-phosphorylation is unstable and heat sensitive to heat. The strain with HK2 overexpression dramatically reduced the intensity of the top band corresponding to phosphorylated Rrp2 (Figure 5, last lane), whereas the strain carrying the same shuttle vector that overexpressed an unrelated protein HD-GYP did not affect the level of Rrp2 phosphorylation (Figure 5, lane 4). This result suggests that although HK2 is not required for Rrp2 phosphorylation, it can function as a phosphatase to dephosphorylate Rrp2, and the impaired activation of σ^54^-σ^S^ cascade by HK2 overexpression is, at least in part, due to the reduced level of Rrp2 phosphorylation.

**Figure 6:**
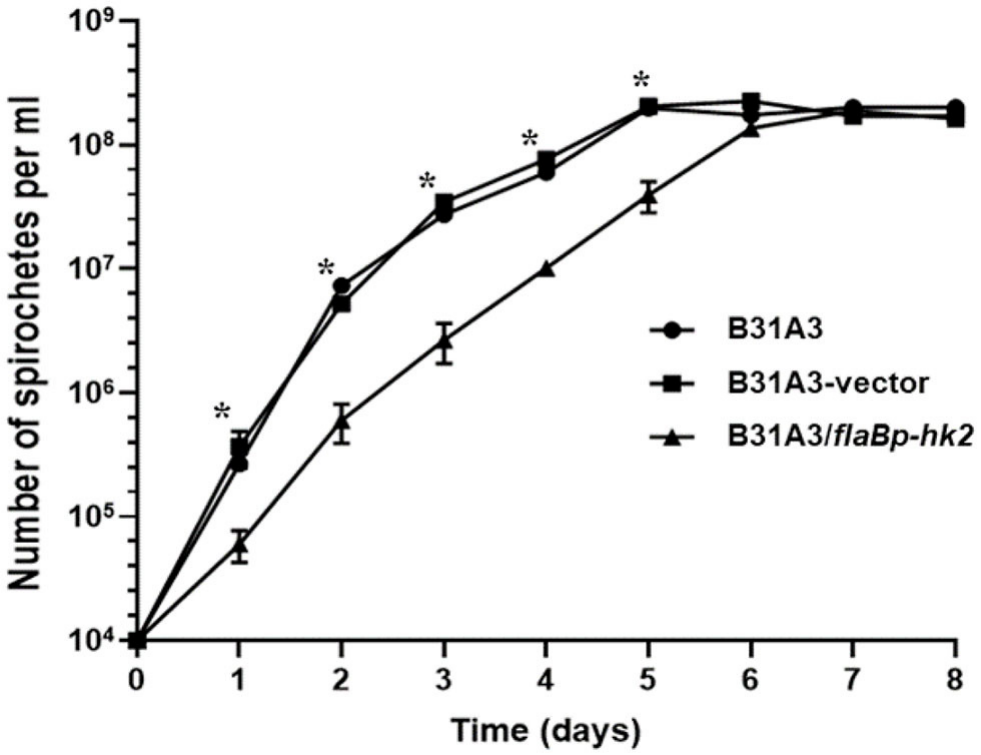
HK2 overexpression resuls in reduced growth rate. Wild-type *B. burgdorferi* strain B31A3, B31A3 carrying an empty shuttle vector (B31A3-vector), or B31A3 carrying a shuttle vector harboring a *hk2* gene driven by a *flaB* promoter (B31A3*/flaBp-hk2*) were cultivated in standard BSK-II medium at 37°C with a initial cell density of 1 × 10^4^ spirochetes/ml. Numbers of spirochetes were enumerated under a dark-field microscope. Each data point is the average of data from three independent cultures. *p < 0.01 (paired student test).

We and others showed that Rrp2 phosphorylation is required for cell growth, in addition to activation of σ^54^-σ^S^ cascade (34, 35). If HK2 overexpression reduces the level of Rrp2 phosphorylation, one would expect that it will affect spirochetal growth. Indeed, B31A3/*flaBp-hk2* displayed a distinct show growth rate than B31A3 and B31A3 carrying an empty shuttle vector (Figure 6). This observation further supports the hypothesis that HK2 functions as a phosphatase of Rrp2.

## Discussion

The Rrp2-RpoN-RpoS pathway, or the σ^54^-σ^S^ alternative sigma factor cascade, is the most studied regulatory pathway in *B. burgdorferi* (13, 14). It plays a major role in controlling differential gene expression during the process of the spirochetal transmission from ticks to mammals and has thus been called “Gatekeeper” (11). Therefore, understanding how this pathway is activated is important for our understanding of how *B. burgdorferi* migrates between ticks and mammals. It has been perplexing that HK2, being the cognate histidine kinase of Rrp2, showed no effect on activation of σ^54^-σ^S^ cascade *in vitro* and is dispensable for mammalian infection via the route of needle inoculation (27, 28). Given that *B. burgdorferi* has a compact genome and that the *hk2* gene is highly conserved among all *B. burgdorferi* strains including *B. garinii* and *B. afzelii*, it is unlikely that *hk2* is no longer needed for *B. burgdorferi* and is in the process of gene loss through genome reduction. In this study, we showed that strain lacking HK2 reduced infectivity via tick bites, the nature route of infection. Tightly controlled *hk2* expression is also important for mammalian infection, as HK2 overexpression led to reduced infectivity in mice. We also successfully employed the Phos-tag method, which not only allowed us to detect phosphorylated form Rrp2 but also showed that although HK2 is not required for Rrp2 phosphorylation *in vitro*, it can function as a phosphatase that dephosphorylates Rrp2. Together, this study demonstrates that HK2 is not what was previously perceived dispensable for the pathogenesis of *B. burgdorferi*; rather, it plays an important role in the enzootic cycle of *B. burgdorferi*.

The observation that the *hk2* mutant showed different infection outcomes between needle inoculation vs. tick bite underlines the importance of using nature route of tick infestation for assessment of infectivity of a *B. burgdorferi* mutant. It has been reported that different route of infection by *B. burgdorferi* can have different infectivity and tissue tropism (36, 37). For example, the *dbpBA* mutant lacking decorin-binding proteins A and B showed avirulent phenotype by needle-inoculation, but later was demonstrated to be fully infectious via tick infestation (36). One of the obvious reasons for such different outcomes is that spirochetes cultivated *in vitro* used for needle inoculation have different gene expression profile from that in ticks. In this regard, we examined the *ospC* expression of the *hk2* mutant during tick feeding, as *ospC* expression is the surrogate for activation of σ^54^-σ^S^ cascade (Figure 2). This is important because the previous conclusion that HK2 is not required for Rrp2-RpoS-RpoS activation is based on spirochetes grown *in vitro*. Whether HK2 plays a role in Rrp2-RpoS-RpoS activation *in vivo* has not been examined. Based on the result from the current study, we now can conclude that HK2 is not essential for Rrp2-RpoS-RpoS activation *in vivo*, i.e., during tick feeding. This data also indicates that the reduced infectivity of the *hk2* mutant via tick bite was not due to a defect in activation of σ^54^-σ^S^ cascade, suggesting HK2 may influence other pathways or genes. For instance, HK2 may regulate expression of genes important for spirochetal migration from the tick midgut to salivary gland, whereas needle inoculation bypasses such requirement.

Our results show that Hk2 can function as a phosphatase to dephosphorylate Rrp2, which could explain why Hk2 overexpression resulted in an impaired activation of σ^54^-σ^S^ cascade and reduced infectivity in mice. However, this observation does not exclude other effects of HK2 overexpression that might also contribute to the phenotype. For example, HK2 overexpression might sequester potential HK2 binding ligand or interfere the interacting partner of Rrp2 such as RpoN and possibly BosR. One caveat of this study is that the phenotype of HK2 overexpression in ticks was not examined. B31A3/*flab-hk2* is defective in mice, which hampered us feeding ticks on infected mice. Further study using artificial feeding to infect ticks is warranted to confirm the HK2 overexpression phenotype in ticks. Nevertheless, although a lot needs to be learned about HK2 function, this work demonstrates, for the first time, that the *hk2* mutant is defective in the enzootic cycle of *B. burgdorferi*, and HK2 can function as a phosphatase for Rrp2. That is, despite the fact that HK2 is not required for Rrp2 phosphorylation *in vitro*, HK2 is important to the enzootic cycle of *B. burgdorferi* and further studies are warranted to elucidate the function of HK2 including the role of the putative PAS domain, the signal HK2 may senses, and the nature of the defect of the *hk2* mutant in the enzootic cycle.

## Materials and Methods

### *B. burgdorferi* Strains and Culture Conditions

Low-passage, virulent *B. burgdorferi* strain B31A3 (a gift from Dr. Patricia Rosa, Rocky Mountain Laboratories, NIH) was used in this study. A B31A3 derived *hk2* mutant used in this study was constructed previously by our laboratory (28). Spirochetes were cultivated in Barbour-Stoenner-Kelly (BSK-II) medium supplemented with 6% normal rabbit serum (Pel-Freez Biologicals, Rogers, AR) (38) at 37°C with 5% CO_2_. For the HK2 overexpression *B. burgdorferi* strain, 300 μg/ml of kanamycin antibiotics was added to the cultures. The constructed shuttle vector (pHX55-HK2) was maintained in *Escherichia coli* strain DH5α.

### Construction of the Strain With Overexpression of *hk2*

For *hk2* overexpression, the PCR fragments of the wild-type *hk2* gene and the *flaB* promoter were fused at the ATG site, and the combined fragment was then cloned into the *BamH*1 and *Pst*I sites of the shuttle vector pJD55 (36), resulting in pHX55-HK2. The constructed shuttle vector was then transformed into B31A3, and kanamycin-resistant *Borrelia* transformants were confirmed by PCR for the presence of pHX55-HK2 and by Western blot for HK2 overproduction. Plasmid profiles of the confirmed transformants were determined by multiple PCR analyses for each of the endogenous plasmids as described previously (39, 40). One of the HK2 overexpression clones that had plasmid profiles identical to parental B31A3 was chosen for further study.

### Mouse Infection With *B. burgdorferi* via Needle Inoculation

All mouse experiments were approved by the IACUC committee of Indiana University School of Medicine (IUSM) under the protocol number #11339. Four-week-old C3H/HeN mice (Harlan, Indianapolis, IN) were subcutaneously inoculated with doses of spirochetes as indicated. Mice were euthanized at the end of the experiments, and multiple tissues (joint, heart, skin) were harvested. All tissues were cultivated in 2 ml of the BSK-II medium (Sigma-Aldrich, St. Louis, MO) containing an antibiotic mixture of phosphomycin (2 mg/ml), rifampin (5 mg/ml), and amphotericin B (250 mg/ml) (Sigma-Aldrich) to inhibit bacterial and fungal contamination. All cultures were maintained at 37°C and examined for the presence of spirochetes by dark-field microscopy beginning from 5 days after inoculation. A single growth-positive culture was used as the criterion to determine positive mouse infection.

### Tick-Mouse Cycle of *B. burgdorferi*

*Ixodes scapularis* egg masses were purchased from Oklahoma State University. The tick-mouse experiments were conducted in IUSM and approved were approved by the IACUC committee of IUSM under protocol number #11339. Unfed larvae were fed on groups of mice (C3H/HeN, three mice per group, 150–200 larvae per mouse) that were needle infected with spirochetes. Ticks were allowed to feed to repletion (3–4 days) and then collected within 24 h. A portion of fed larvae were subjected to analyses. The remaining fed larvae were maintained in the tick incubator and allowed to molt to the nymphal stage (about 5 weeks). One month after molting, unfed nymphs were then allowed to feed on naive C3H/HeN mice. Fully engorged nymphal ticks were collected within 24 h of repletion and subjected to analyses. Mice infected with tick bites were subjected to infection analyses as described above.

### Immunofluorescence Assay (IFA)

IFA was performed as reported previously (3). Briefly, the entire contents of a fed tick were smeared and fixed on silylated microscope slides (CEL Associates, Pearland, TX). The slides were incubated with BacTrace fluorescein isothiocyanate-conjugated goat anti-*B. burgdorferi* antibody (Kirkegaard and Perry Laboratories Gaithersburg, MD) at 37°C. Samples were observed using an Olympus BX50 fluorescence microscope. Twenty ticks from each group were examined by IFA.

### qPCR and qRT-PCR

For qPCR analyses of *B. burgdorferi* DNA in ticks, DNA samples were extracted from ticks using a DNeasy tissue kit (Qiagen, Valencia, CA) according to the manufacturer's protocol. qPCR was performed with primer pairs of qflaB-F/R and qTactin-F/R as described previously (41). Calculations of relative DNA copy number (represented by *flaB*) were normalized with the copy number of the tick actin gene.

For quantification of *ospC* transcripts of *B. burgdorferi* in ticks, RNA samples were extracted from ticks using the RNeasy mini kit (Qiagen, Valencia, CA) according to the manufacturer's protocols. To reduce trace amounts of DNA contamination, samples were further digested with RNase-free DNaseI (Qiagen), purified using the RNeasy mini kit (Qiagen), and analyzed with NanoDrop One^C^ Spectrophotometer (Thermo Fisher Scientific). DNA-free RNA was confirmed by PCR amplification for the *B. burgdorferi flaB* gene. cDNA was synthesized using the PrimeScript 1st strand cDNA Synthesis Kit (TaKaRa). Given the low levels of bacterial RNA in ticks, the specific primers for each gene target were used for cDNA synthesis instead of random primers previously (41). To quantify the transcript levels of genes of interest, an absolute quantitation method was used to create a standard curve for the qPCR assay according to the manufacturer's protocol (Strategene, La Jolla, CA). Briefly, the PCR product of the *flaB* gene served as a standard template. A series of 10-fold dilutions (10^2^-10^7^ copies/ml) of the standard template was prepared, and qPCR was performed to generate a standard curve by plotting the initial template quantity against the Ct values for the standards. The quantity of the targeted genes in the cDNA samples was calculated using their Ct values and the standard curve. The samples were assayed in triplicate using the ABI 7000 Sequence Detection System and PowerUp SYBR Green Master Mix (Applied Biosystems). The levels of the target gene transcript were reported as per 1000 copies of *flaB*.

For qRT-PCR analyses of gene expression in cultured *B. burgdorferi*, spirochetes were cultured at 37°C in BSK-II medium and harvested in the late-log phase. RNAs were extracted and subjected to qRT-PCR analyses as described above. Primers used for *rpoS, ospC, bb0680, bb0844, bba07, bba73* were described previously (42). Samples were first normalized with the *flaB* level, and then levels of gene expression were reported relative to that of wild-type B31A3 (with the level of expression of each gene in B31A3 as 1.0).

### Sodium Dodecyl Sulfate-Polyacrylamide Gel Electrophoresis (SDS-PAGE) and Immunoblotting

Spirochetes from mid-log cultures were harvested by centrifugation at 8,000 × g for 10 min and washed three times with PBS (pH 7.4) at 4°C. Pellets were suspended in SDS buffer containing 50 mM Tris-HCl (pH 8.0), 0.3% sodium dodecyl sulfate (SDS), and 10 mM dithiothreitol (DTT). Cell lysates (10^8^ cells per lane) were separated by 12% SDS-polyacrylamide gel electrophoresis (PAGE) and transferred to nitrocellulose membranes (GE-Healthcare, Milwaukee, WI). Membranes were blotted with either single or a mixed monoclonal/polyclonal antibodies against HK2 (28), RpoS, OspC, DbpA, BBK32 (43), followed with goat anti-mouse or anti-rat lgG-HRP secondary antibody (1:1,000; Santa Cruz Biotechnology). Detection of horseradish peroxidase activity was determined by the enhanced chemiluminescence method (Thermo Pierce ECL Western Blotting Substrate) with subsequent exposure to X-ray film.

### Phos-Tag SDS-PAGE

Spirochetes were grown at mid-log phase and harvested by centrifugation for 1 min at 4°C. Cell pellets were washed twice with ice-cold PBS and resuspended in 1 x SDS-PAGE sample buffer. To get rid of the cell debris, the samples were centrifuged at 4°C for 3 min. The supernatants were then loaded on 7.5% Phos-tag SDS-PAGE gels with or without Phos-tag (33). Phos-tag acrylamide AAL-107 was purchased from Wako Chemicals USA. To prepare the control sample in which all Rrp2 molecules are dephosphorylated, cell lysates were boiled for 5 min prior to loading into Phos-tag gel. The gels were run in MOPS buffer (100 mM Tris-HCl, 100 mM MOPS, 0.1% SDS, and 5 mM sodium bisulfite) at 100V, 4°C followed by treatment with transfer buffer (25 mM Tris-HCl, 192 mM glycine, 20% methanol) containing 1 mM EDTA at room temperature with gentle shaking for 15 min to remove the zinc from the gel. The gel was further washed in new transfer buffer without EDTA for 15 min at room temperature with gentle shaking. Separated proteins in the gels were transferred onto NC or PVDF membranes for immunoblot.

## Data Availability Statement

The original contributions presented in the study are included in the article/supplementary material, further inquiries can be directed to the corresponding authors.

## Ethics Statement

The animal study was reviewed and approved by the IACUC committee of Indiana University School of Medicine (IUSM) under the protocol number # 11339.

## Author Contributions

QL and HX performed the experiment and wrote the paper. YZha and JY performed the experiment. JD performed the experiment and data analysis. YZho analyzed the data and edits the paper. XY and YL designed and wrote the paper. All authors contributed to the article and approved the submitted version.

## Conflict of Interest

The authors declare that the research was conducted in the absence of any commercial or financial relationships that could be construed as a potential conflict of interest.
