# Corrigendum: Role of HK2 in the Enzootic Cycle of *Borrelia burgdorferi*

**DOI:** 10.3389/fmed.2021.668709

**Published:** 2021-03-31

**Authors:** Qiang Liu, Haijun Xu, Yan Zhang, Jing Yang, Jimei Du, Yan Zhou, X. Frank Yang, Yongliang Lou

**Affiliations:** ^1^Wenzhou Key Laboratory of Sanitary Microbiology, Key Laboratory of Laboratory Medicine, Ministry of Education, School of Laboratory Medicine and Life Sciences, Wenzhou Medical University, Wenzhou, China; ^2^Department of Microbiology and Immunology, Indiana University School of Medicine, Indianapolis, IN, United States; ^3^State Key Laboratory of Rice Biology and Ministry of Agriculture Key Laboratory of Agricultural Entomology, Institute of Insect Sciences, Zhejiang University, Hangzhou, China; ^4^Optometry and Eye Hospital and School of Ophthalmology, School of Biomedical Engineering, Wenzhou Medical University, Wenzhou, China

**Keywords:** lyme disease, *Borrelia* (Borreliella) *burgdorferi*, two-component system (TCS), Rrp2-HK2, OspC

In the original article, there was a mistake in Figure 5 as published. Due to an error in compiling multi-panel images, a gap between the image of “B31-A3” and the image of “B31A3/flaBp-HD-GYP; B31A3/flaBp-hk2” was omitted. In this figure, unphosphorylated Rrp2 (lower lane) serves as an internal control for each sample. Overproduction of an unrelated protein HD-GYP (B31A3/flaBp-HD-GYP) serves as the negative control for overproduction of Hk2 (B31A3/flaBp-hk2), showing a reduction of Rrp2 phosphorylation by overexpression of Hk2. The corrected Figure 5 appears below.

**Figure 5:**
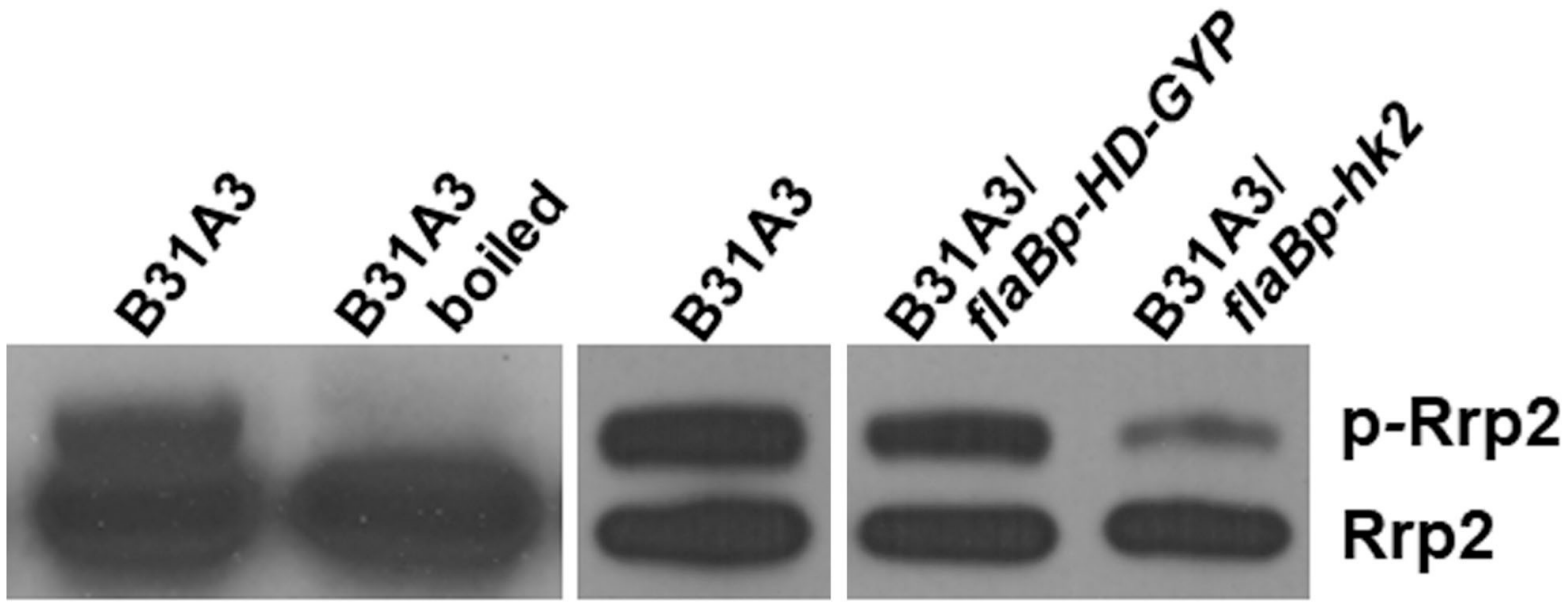
Overexpressing HK2 reduces the level of phosphorylated Rrp2 in *B. burgdorferi*. Phos-tag SDS-PAGE and immunoblotting was used to detect both phosphorylated and dephosphorylated Rrp2 in the cell. Wild-type *B. burgdorferi* B31A3, B31A3 carrying a shuttle vector harboring a unrelated protein HD-GYP (B31A3*/flaBp-HD-GYP*), or B31A3 carrying a shuttle vector harboring a *hk2* gene driven by a *flaB* promoter GYP (B31A3*/flaBp-hk2*), were harvested at mid-log phase and cell lysates were prepared and separated on 7.5% SDS-PAGE containing 0, 5, 10, and 25 uM Phos-tag followed by immunoblotting using anti-Rrp2 antibody. p-Rrp2, the band corresponds to phosphorylated Rrp2. As a unphosphorylated Rrp2 control, B31A3 was also treated by boiling (lane 2) prior to Phos-tag SDS-PAGE (Rrp2 phosphorylation is unstable and sensitive to heat).

The authors apologize for this error and state that this does not change the scientific conclusions of the article in any way. The original article has been updated.

